# A phenotypic screen of the *Global Health Priority Box* identifies an insecticide with anthelmintic activity

**DOI:** 10.1186/s13071-024-06183-y

**Published:** 2024-03-14

**Authors:** Harrison T. Shanley, Aya C. Taki, Joseph J. Byrne, Nghi Nguyen, Tim N. C. Wells, Abdul Jabbar, Brad E. Sleebs, Robin B. Gasser

**Affiliations:** 1 Department of Veterinary Biosciences, Melbourne Veterinary School, Faculty of Science, The University of Melbourne, Parkville, VIC 3010 Australia; 2 Walter and Eliza Hall Institute of Medical Research, Parkville, VIC 3052 Australia; 3 Medicines for Malaria Venture (MMV), 1215 Geneva, Switzerland

**Keywords:** Anthelmintics, Drug discovery, Nematodes, Antiparasitics, Phenotypic screening, *Haemonchus contortus*, *Caenorhabditis elegans*, Flufenerim, Global Health Priority Box

## Abstract

**Background:**

Infection with parasitic nematodes (helminths), particularly those of the order Strongylida (such as *Haemonchus contortus*), can cause significant and burdensome diseases in humans and animals. Widespread drug (anthelmintic) resistance in livestock parasites, the absence of vaccines against most of these nematodes, and a lack of new and effective chemical entities on the commercial market demands the discovery of new anthelmintics. In the present study, we searched the Global Health Priority Box (Medicines for Malaria Venture) for new candidates for anthelmintic development.

**Methods:**

We employed a whole-organism, motility-based phenotypic screening assay to identify compounds from the Global Health Priority Box with activity against larvae of the model parasite *H. contortus*, and the free-living comparator nematode *Caenorhabditis elegans*. Hit compounds were further validated via dose–response assays, with lead candidates then assessed for nematocidal activity against *H. contortus* adult worms, and additionally, for cytotoxic and mitotoxic effects on human hepatoma (HepG2) cells.

**Results:**

The primary screen against *H. contortus* and *C. elegans* revealed or reidentified 16 hit compounds; further validation established MMV1794206, otherwise known as ‘flufenerim’, as a significant inhibitor of *H. contortus* larval motility (half-maximal inhibitory concentration [IC_50_] = 18 μM) and development (IC_50_ = 1.2 μM), *H. contortus* adult female motility (100% after 12 h of incubation) and *C. elegans* larval motility (IC_50_ = 0.22 μM). Further testing on a mammalian cell line (human hepatoma HepG2 cells), however, identified flufenerim to be both cytotoxic (half-maximal cytotoxic concentration [CC_50_] < 0.7 μM) and mitotoxic (half-maximal mitotoxic concentration [MC_50_] < 0.7 μM).

**Conclusions:**

The in vitro efficacy of MMV1794206 against the most pathogenic stages of *H. contortus*, as well as the free-living *C. elegans*, suggests the potential for development as a broad-spectrum anthelmintic compound; however, the high toxicity towards mammalian cells presents a significant hindrance. Further work should seek to establish the protein–drug interactions of MMV1794206 in a nematode model, to unravel the mechanism of action, in addition to an advanced structure–activity relationship investigation to optimise anthelmintic activity and eliminate mammalian cell toxicity.

**Graphical Abstract:**

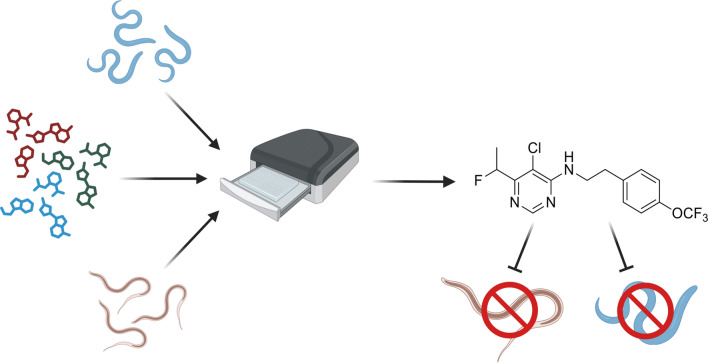

## Background

Infections and diseases (nematodiases) caused by gastrointestinal parasitic nematodes are a significant strain on both human and animal health [1–3]. The control of soil-transmitted helminths (STHs) was included in the World Health Organization (WHO)’s neglected tropical disease road map for 2021–2030 [3], with the core strategic interventions and critical actions focused on adequate hygiene, education, preventative chemotherapy and the development of more effective medicines in the case of emerging drug resistance [3]. In 2019, ~ 909 million people were estimated to be infected with intestinal nematodes (*Ascaris, Trichuris, Ancylostoma* and *Necator*), accounting for 1.97 million disability-adjusted life years [1], disproportionately affecting communities facing poverty. On the other hand, livestock helminth infections are ubiquitous, with parasites such as *Haemonchus, Cooperia, Ostertagia, Teladorsagia* and *Trichostrongylus* impacting producers globally [2, 4, 5]. In Australia alone, the annual cost due to nematodiases (in sheep and cattle) is estimated at USD 450 million [5]—a significant economic burden. Globally, the market for anti-parasitic drugs for livestock animals is estimated at USD 2.5 billion per annum [4], indicative of the impact of parasitic worms.

Central to the management of such parasites are knowledge and understanding of epidemiology (reviewed by [6, 7]), regular monitoring and accurate diagnoses of infections [8–10] and implementation of an anthelmintic regimen to achieve control. Although vaccination would be a preferred method to prevent infections and disease, vaccines have been challenging to develop [11], and only two have been commercialised for use in livestock (Bovilis Huskvac^®^ and Barbervax^®^ to prevent bovine dictyocaulosis and haemonchosis, respectively). Thus, anthelmintic treatment remains a key component of most control programs. However, unfortunately, drug resistance in nematodes of livestock is now widespread due to the excessive and/or widespread use of commercially available anthelmintics [2, 12–17]. As monepantel (Zolvix™) [18] and derquantel (Startect^®^) [19–21] are the only new anthelmintic drug classes (for use in livestock) introduced into the commercial market since 2000, there is an urgent need to discover and develop new anthelmintic compounds with novel mechanisms/modes of action.

Historically, anthelmintic drug discovery has been hindered by a lack of adequate screening/drug development technologies, and the economic costs associated with the drug discovery process (reviewed by [22, 23]). Early drug development campaigns relied on the testing of anthelmintic candidates in animal models [24–27]. Whilst effective, these assays are low-throughput, high-cost and labour-intensive. Today, anthelmintic drug discovery focuses on the use of in vitro methods to quantitatively assess test compounds for antiparasitic activity. A number of practical, time-efficient and relatively low-cost phenotypic (whole-worm) screening methods have been developed for the discovery of novel anthelmintic candidates. Methods vary in the species assessed, life cycle stage used, technology used for assessment, phenotypic characteristics assessed and the throughput potential (reviewed by [23]). Medium- to high-throughput assays reported to date utilise heat flow- [28, 29], impedance- [30], imaging- [31–33] or photometry-based [34–39] techniques to evaluate test compounds.

Anthelmintic discovery is being enabled through the use of multi-omic tools and resources for key parasitic nematodes such as *Haemonchus contortus* (order Strongylida; clade V) [40–44] and its free-living relative, *Caenorhabditis elegans* (reviewed by [45, 46]). Drug discovery efforts have also been enhanced by the development of proteomic-driven target deconvolution tools (reviewed by [47, 48]), allowing for the identification of novel drug–protein interactions. Thus, early-stage broad-spectrum nematocidal development may be achieved, first, through the discovery of new drug candidates via a phenotypic screening platform (utilising one or more model nematode organisms), and subsequent target deconvolution using advanced workflows, assisted by bioinformation.

In the further pursuit of a new and effective anthelmintic entity, together with the Medicines for Malaria Venture (MMV; Geneva, Switzerland), we previously screened a number of small compound collections, including the Pathogen, Stasis, and Pandemic Response Boxes [49–51] for anthelmintic activity against *H. contortus* and/or *C. elegans*. Here, as part of an ongoing collaboration with MMV, we screened the recently curated Global Health Priority Box in a whole-organism, motility-based phenotypic assay [38, 39] for anthelmintic activity on the larvae *H. contortus* and *C. elegans*. This Box contains 240 compounds which are at various stages of development and have demonstrated activity against drug-resistant *Plasmodium* (*n* = 80 entities), neglected and zoonotic pathogens (*n* = 80) and various vectors (such as species of mosquitoes, ticks and mites) (*n* = 80). Then, we estimated the anthelmintic potency of (hit) compounds, evaluated the toxicity of hit compounds against HepG2 cells and worked toward identifying candidates for future medicinal chemistry optimisation and mechanism/mode of action studies.

## Methods

### Preparation of compounds for screening

The Global Health Priority Box compound collection from MMV, Geneva, Switzerland, contains 240 chemical entities, at various stages of development, with activity against drug-resistant *Plasmodium*, neglected and zoonotic pathogens (such as *Leishmania, Mycobacterium* and *Trypanosoma*) and vectors (such as species of mosquitoes, ticks and mites) (https://www.mmv.org/mmv-open/global-health-priority-box/about-global-health-priority-box; accessed 18 September 2023). Individual compounds were supplied as solid samples; each compound was resuspended in 10 μl of (100%) dimethyl sulfoxide (DMSO) to give a final concentration of 10 mM. Prior to screening, test compounds were each diluted to 40 μM in sterile lysogeny broth (LB; cf. [38, 52]) containing 100 IU/ml of penicillin, 100 µg/ml of streptomycin and 0.25 µg/ml of amphotericin B (Fungizone^®^, Thermo Fisher Scientific, Waltham, MA, USA); this supplemented LB was designated as LB*.

### Production, storage and preparation of *H. contortus* larvae and adults

*Haemonchus contortus* (Haecon-5 strain; cf. [53]) was produced in experimental sheep as described previously [54] and in accordance with the institutional animal ethics guidelines (permit no. 23983-2811-4; The University of Melbourne, Parkville, Victoria, Australia). Helminth-free Merino sheep (6 months of age; male) were orally inoculated with 7000 third-stage larvae (L3s) of *H. contortus*. Four weeks after inoculation, faecal samples were collected from sheep with patent *H. contortus* infection. These samples were incubated at 27 °C and > 90% relative humidity for 1 week to yield larvae [54], which were then collected in tap water and allowed to migrate through two layers of nylon mesh (pore size: 20 μm; Rowe Scientific, Doveton, Victoria, Australia) to remove debris. Clean L3s were stored in the dark at 11 °C for up to 6 months [54]. Immediately prior to use in assays, *H. contortus* L3s were artificially exsheathed via exposure to 0.15% (*v*/*v*) sodium hypochlorite for 20 min at 38 °C [54], achieving an exsheathment rate of 90%. The larvae were then immediately washed five times with 50 ml of sterile physiological saline solution by centrifugation at 2000×*g* (5 min) and resuspended in LB* at a concentration of 200 xL3s per 50 µl (for the primary screen) or 300 xL3s per 50 µl (for the dose–response assays).

Adult *H. contortus* were collected from the abomasa of sheep infected for 10 weeks, then washed extensively in RPMI 1640 media supplemented with final concentrations of 2 mM L-glutamine, 100 IU/ml of penicillin, 100 µg/ml of streptomycin and 0.25 µg/ml of amphotericin B (Thermo Fisher Scientific, Scoresby, VIC, Australia; this supplemented RPMI was designated as RPMI*). Using a dissecting microscope, female and male worms were separated (38 °C in RPMI*) immediately prior to compound testing.

### Production, storage and preparation of *C. elegans* larvae

*Caenorhabditis elegans* (N2—wild-type Bristol strain) was maintained in the laboratory under standard conditions at 20 °C on nematode growth media (NGM) agar plates, with *Escherichia coli* OP50 as a food source (Stiernagle, 2006). Gravid adult worms were collected from NGM plates, washed with sterile M9 buffer and then treated with a solution containing 0.4% (*v*/*v*) sodium hypochlorite and 170 mM sodium hydroxide for 4–8 min at 22–24 °C (room temperature) to release eggs [55, 56]. The eggs were then washed five times with 15 ml of sterile M9 buffer (centrifugation at 500×*g*, 2 min). After washing, the egg pellet was suspended in 8 ml of M9 buffer in a 15 ml tube and gently agitated for 24 h at 22–24 °C to produce first-stage larvae (L1s); 45 h prior to screening, synchronised *C. elegans* L1s were inoculated onto NGM plates containing 500 µl of *E. coli* OP50 (~ 3000 larvae per plate) and allowed to develop to fourth-stage larvae (L4s) at 20 °C. L4s were collected from plates and washed twice with sterile M9 buffer by centrifugation (500×*g*, 2 min) to remove *E. coli* OP50, and then resuspended in LB* at a concentration of 125 L4s per 50 µl (for the primary screen) or 100 L4s per 50 µl (for the dose–response assays).

### Screening for anthelmintic activity against *H. contortus* larvae

An established high-throughput phenotypic screening assay [38] was used to test the anthelmintic activity of compounds on *H. contortus* xL3s. Compounds were assessed for motility inhibition at a concentration of 20 µM in LB*. Four compounds—monepantel (Zolvix™; Elanco, Australia), monepantel/abamectin (Zolvix Plus™; Elanco, Australia), moxidectin (Cydectin^®^; Virbac, France) and compound MIPS-0018666 (abbreviated herein as M-666; cf. [57])—were used as positive controls (final concentration of 20 µM in LB*). A solution of LB* + 1% (*v*/*v*) DMSO was used as a negative control. Test compounds were distributed amongst one flat-bottom, 384-well microplate (cat no. 3860; Corning, Corning, NY, USA). Eighty xL3s of *H. contortus* in 20 µl of LB* were added to each well to give a final volume of 40 µl. The plate was then placed in a CO_2_ incubator (10% [*v*/*v*] CO_2_, 38 °C, > 90% humidity). At 90 h, worm activity was captured using a WMicroTracker ONE unit (Phylumtech, Sunchales, Santa Fe, Argentina). Over a period of 15 min, disturbance of an infrared beam in individual wells was recorded as a worm ‘activity count’. Activity counts were then normalised to the positive and negative controls using the GraphPad Prism program (v.9.1.0 GraphPad Software, San Diego, CA, USA). The screening plate was then returned to the incubator (10% [*v*/*v*] CO_2_, 38 °C, > 90% humidity) for an additional 72 h. At 168 h, worms were fixed with 40 µl of Lugol solution (Sigma-Aldrich, St. Louis, MO, USA). Compounds were then assessed (microscopically) for inhibition of larval development and/or induction of a non-wild-type phenotype in *H. contortus* worms. A compound that reduced xL3 motility by ≥ 70% and/or inhibited larval development or induced an abnormal phenotype (relative to the negative control) was recorded as a ‘hit’ compound. The performance of the assay was monitored using the Z′-factor [58] calculated using data for the negative (DMSO) and positive (M-666) control compounds on individual plates.

### Screening for anthelmintic activity against *C. elegans*

An established assay [39] was employed to test the anthelmintic activity of compounds on *C. elegans*. Test compounds and positive and negative controls were prepared in a flat-bottom, 384-well microplate (cf. [39]). Fifty L4s of *C. elegans* in 20 µl of LB* were added to each well to give a final volume of 40 µl. The plate was then placed in an incubator (Thermo Fisher Scientific, Waltham, MA, USA) at 20 °C for 40 h. At 40 h, the worm (in transition from L4s to young adults) activity was captured (over a period of 15 min) utilising the WMicroTracker ONE unit. Activity counts were then normalised to the positive and negative controls using the GraphPad Prism software program (v.9.1.0). A compound that reduced worm motility by ≥ 70% (relative to the negative control) was recorded as a hit compound. The Z′ factor was calculated in the same manner as described previously [58].

### *Haemonchus contortus* dose–response assay

The dose–response assay for *H. contortus* followed a well-established protocol [38]; it was employed to evaluate the potency of hit compounds against this nematode. Test compounds were assessed individually for an effect on the motility of xL3s (10-point, twofold serial dilution in LB*, 40–0.16 μM). One compound, monepantel (prepared in the same manner as the test compounds), was used as a positive control. A solution of LB* was used as a negative control. The test and positive-control compounds were arrayed in triplicate across individual flat-bottom 96-well microplates, with six wells on each plate containing the negative control. Three hundred xL3s of *H. contortus* in 50 μl of LB* were added to each well to give a final volume of 100 μl. Plates were then placed in a CO_2_ incubator (10% [*v*/*v*] CO_2_, 38 °C, > 90% humidity). After 168 h of incubation, worm activity was captured using a WMicroTracker ONE unit. Over a period of 15 min, disturbance of an infrared beam in individual wells was recorded as a worm activity count. Raw activity counts for each well were normalised to the negative controls. The compound concentrations were log_10_-transformed and fitted using a variable-slope four-parameter equation, using the ordinary least squares fit model, employing GraphPad Prism (v.9.1.0). Larval development was established at 168 h of incubation with compound, as described previously [54]. The development inhibition and phenotypes of larvae were examined microscopically [54]. A one-way analysis of variance (ANOVA) with a Tukey multiple comparison test or an unpaired *t*-test was used to establish statistically significant differences in larval motility or development.

### *Caenorhabditis elegans* dose–response assay

The dose–response assay for *C. elegans* followed a well-established protocol [39] and was employed to evaluate the potency of hit compounds against this nematode. Test and positive-control compounds as well as negative controls were prepared in 96-well microplates (cf. [39]). One hundred *C. elegans* in 50 μl of LB* were added to each well to give a final volume of 100 μl. Plates were then placed in an incubator at 20 °C for 40 h. At 40 h, worm activity was captured using a WMicroTracker ONE unit. Raw activity counts for each well were normalised to the negative controls. The compound concentrations were log_10_-transformed and fitted using a variable-slope four-parameter equation, using the ordinary least squares fit model, employing GraphPad Prism (v.9.1.0). A one-way ANOVA with a Tukey multiple comparison test or an unpaired *t*-test was used to establish statistically significant differences in larval motility.

### Assessment of the activity of selected compounds on *H. contortus* adults

The activity of two test compounds was assessed on adult female specimens of *H. contortus* in an established assay [59]. The compound was added in triplicate to the wells of a 24-well plate (Corning, USA) at a concentration of 40 μM in 500 μl of RPMI*. Two positive-control compounds, monepantel and moxidectin, and a negative control containing 1% (*v*/*v*) DMSO only were included in triplicate on the same plate. Three adult females were added to each of the triplicate wells containing either the test compound or the controls and placed in a CO_2_ incubator (10% [*v*/*v*] CO_2_, 38 °C, > 90% relative humidity) for 24 h. A video recording (30 s) of each well was taken at 3 h, 6 h, 12 h and 24 h during the total incubation period to assess the reduction in worm motility, which was scored as 3 (‘good’), 2 (‘low’), 1 (‘very low’) or 0 (‘no movement’; cf. [59]). For each test or control compound, the motility scores for each of the triplicate wells were calculated, normalised with reference to the negative control (100% motility) and recorded as a percentage. A two-way ANOVA with a Tukey multiple comparison test was used to establish statistically significant differences in worm motility.

### Evaluation of the cellular and mitochondrial toxicity using HepG2 cells

The cytotoxic and mitotoxic activity of MMV1794206 on HepG2 human hepatoma cells was evaluated using an established protocol [60–63]. The test compounds were serially diluted (seven-point, twofold serial dilution, 40–0.63 µM) in Dulbecco’s modified Eagle medium (DMEM; Thermo Fisher Scientific, USA), with GlutaMax™ supplemented with 25 mM D-glucose (cytotoxicity) or D-galactose (mitotoxicity), 10% heat-inactivated foetal bovine serum (FBS), 100 IU/ml of penicillin, 100 µg/ml of streptomycin and 0.25 µg/ml of amphotericin B (denoted as DMEM*). Monepantel and moxidectin (prepared in the same manner as the test compound) were included as reference compounds. Two compounds, doxorubicin (cytotoxic; Sigma-Aldrich, USA) and M-666 (mitotoxic; [57]), were used as positive controls at a single concentration of 10 µM. A solution of DMEM* + 0.25% (*v*/*v*) DMSO was used as a negative control. HepG2 cells were seeded into wells of a 96-well plate in 80 µl of DMEM* (at 1 × 10^5^ cells per well) and allowed to adhere for 16 h at 37 °C and 5% (*v*/*v*) CO_2_ at > 90% humidity prior to incubation with individual compounds, at a final volume of 100 µl. For the assessment of mitochondrial toxicity, cells were starved of serum (DMEM* without FBS) for 4 h prior to the incubation with compounds [60, 61]. Following 48 h of incubation with compounds, cell viability was determined by crystal violet staining [62]. The absorbance (595 nm) of treated cells was normalised using the negative controls (viability: 100%) to calculate the cell viability. All compounds and controls were tested in triplicate. To determine the half-maximal cytotoxic concentration (CC_50_) and half-maximal mitotoxic concentration (MC_50_) values, compound concentrations were log_10_-transformed, baseline-corrected using a respective positive control (doxorubicin or M-666) and fitted using a variable-slope four-parameter equation and ordinary least squares fit model using GraphPad Prism (v.9.1.0).

## Results

### A primary screen identifies 16 compounds with anthelmintic activity

**Fig. 1:**
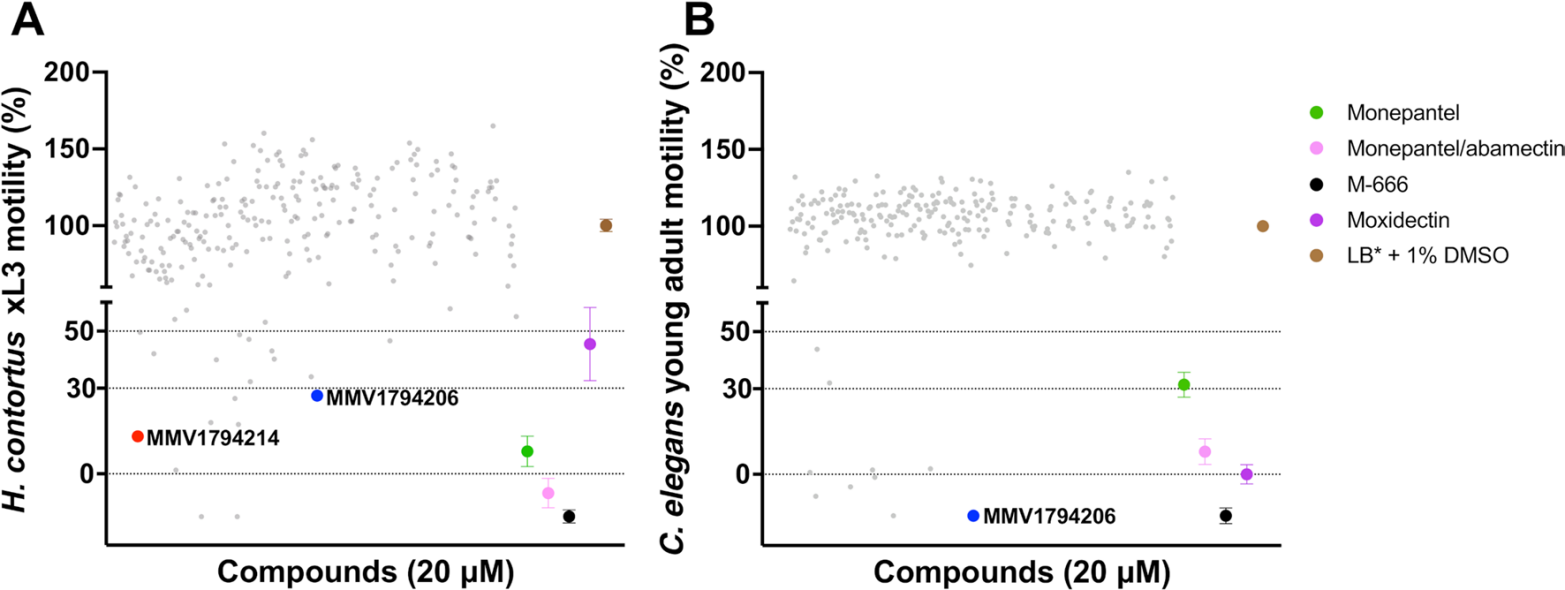
Results of the primary screen of the Medicines for Malaria Venture (MMV) Global Health Priority Box (*n* = 240) against (**A**) exsheathed third-stage larvae (xL3s) of *Haemonchus contortus* and (**B**) young adults of *Caenorhabditis elegans* with reference to four distinct control compounds (monepantel, monepantel/abamectin, M-666 and moxidectin) and a negative (LB* + 1% DMSO) control. All test and positive-control compounds were tested at 20 μM. Each grey dot represents an individual test compound. Mean ± standard error of the mean (SEM) indicated for positive-control compounds (four data points) and negative controls (32 data points for LB* + 1% DMSO). For all screens, the Z′ factor ranged between 0.76 and 0.92

Sixteen compounds (Table 1; Fig. 1A) were shown to significantly inhibit the motility (90 h) and/or development (168 h) of exsheathed L3s (xL3s) of *H. contortus*. MMV1577458, MMV688934, MMV002519 and MMV1794206 reduced larval motility by 72–100% and development by 100%, and each of these compounds induced an abnormal phenotype (*Str* or *Cur*; Table 1). Compounds MMV1794214, MMV1577463 and MMV027339 reduced larval motility by 73–87%, but did not inhibit development or induce an abnormal phenotype. Nine compounds (i.e. MMV1577454, MMV1633829, MMV1578924, MMV1634081, MMV1577467, MMV1633828, MMV672931, MMV1633823 and MMV002231) inhibited larval motility by < 70% after 90 h, and inhibited development by ≥ 90% after 168 h.

**Table 1:** Results of the primary screen of the Global Health Priority Box and four positive-control compounds (monepantel, moxidectin, monepantel/abamectin and M-666) against exsheathed third-stage larvae (xL3s) of *Haemonchus contortus* and young adults of *Caenorhabditis elegans*

Compound	*H. contortus*			*C. elegans*
	Reduction in larval motility; % (SEM)^a^	Larval development inhibition; %^b^	Abnormal phenotype^b,c^	Reduction in young adult motility; % (SEM)^d^
MMV1577458	115 (6)	100	*Cur*	115 (7)
MMV688934	115 (6)	100	*Str*	8.28 (16.0)
MMV002519	98.7 (10.7)	100	*Str*	− 10.8 (14.4)
MMV1794214	86.8 (16.8)	0	–	25.6 (0.1)
MMV1577463	82.7 (0.8)	0	–	− 6.1 (13.5)
MMV027339	73.6 (15.5)	0	–	− 0.9 (16.3)
MMV1794206	72.6 (32.5)	100	*Cur*	115 (7)
MMV1577454	60.0 (34.9)	100	*Str*	98.5 (4.1)
MMV1633829	59.8 (3.2)	90	–	98.1 (22.9)
MMV1578924	57.9 (29.8)	90	–	99.3 (7.2)
MMV1634081	57.0 (4.1)	95	*Str*	− 9.8 (2.6)
MMV1577467	51.2 (15.8)	100	*Str*	21.1 (1.6)
MMV1633828	34.7 (5.1)	100	–	108 (2)
MMV672931	22.0 (16.3)	95	–	104 (12)
MMV1633823	6.98 (35.7)	90	–	68.0 (11.7)
MMV002231	− 27.4 (30.1)	100	*Cur*	101 (19)
Monepantel	92.2 (5.4)	100	*Coi*	68.6 (4.4)
Moxidectin	54.7 (13)	100	–	100 (3)
Monepantel/abamectin	107 (5)	100	*Coi*	92.1 (4.5)
M-666	115 (3)	100	*Cur*	115 (3)

^a^Assessed after 90 h of incubation with a single concentration (20 µM) of compound, calculated from two independent assays

^b^Assessed after 168 h of incubation with a single concentration (20 µM) of compound

^c^*Cur*, curved; *Str*, straight; –, no apparent distinction from wild-type; *Coi*, coiled

^d^Assessed after 40 h of incubation with a single concentration (20 µM) of compound, calculated from two independent assays

Eight of the 16 compounds (Table 1; Fig. 1B) were found to have activity against young adults of *C. elegans* (40 h). MMV1577458 and MMV1794206 both inhibited motility by 100%, whereas compounds MMV1633828, MMV672931, MMV002231, MMV1578924, MMV1577454 and MMV1633829 inhibited larval motility in the range of 85–94%. Throughout the primary screens on *H. contortus* and *C. elegans*, the Z′-factor ranged between 0.76 and 0.92.

### Potency and toxicity assessment reveals MMV1794206 as an anthelmintic candidate

**Fig. 2:**
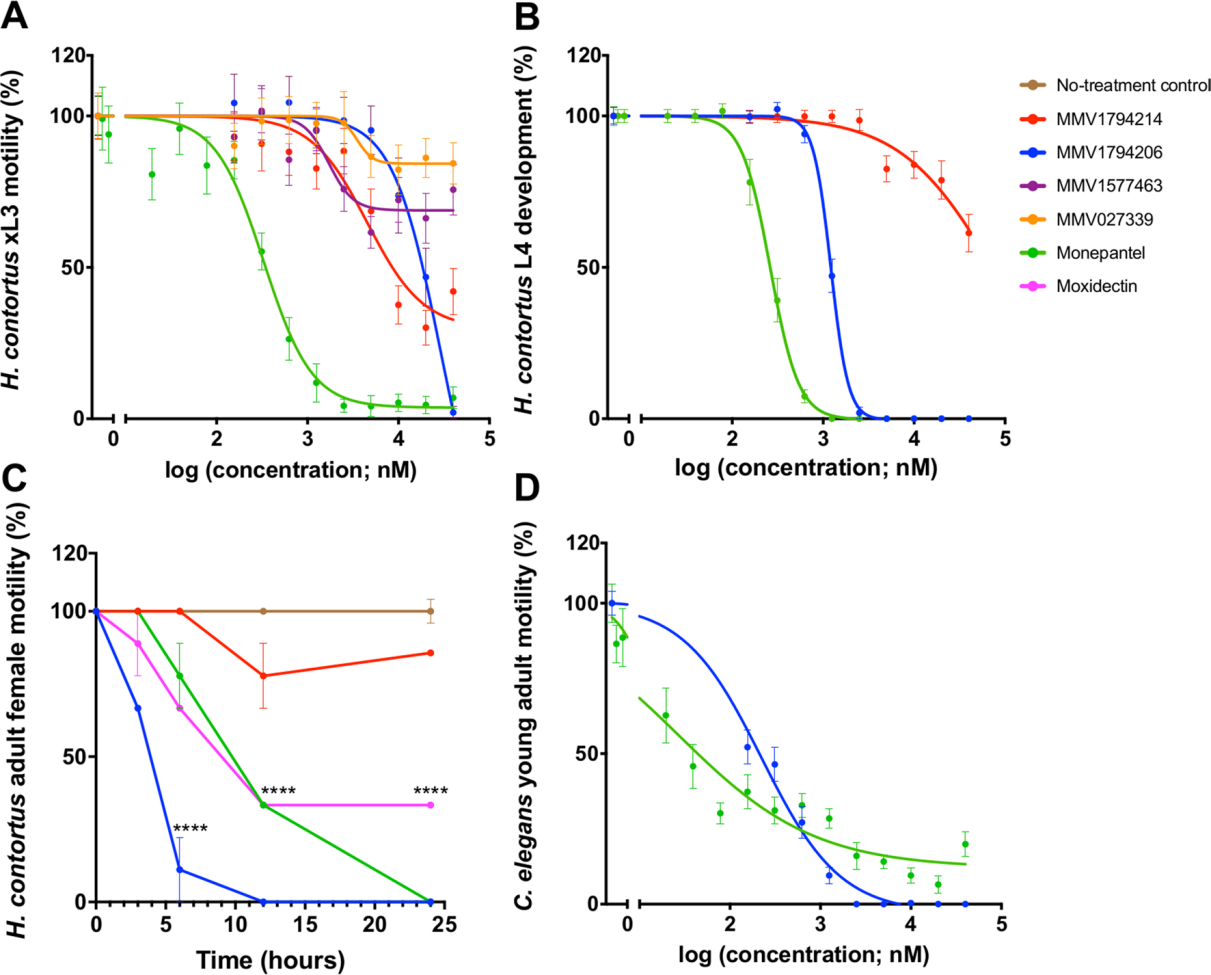
The potency of four active test compounds (MMV1794214, MMV1794206, MMV1577463 and MMV027339) against exsheathed third-stage larvae (xL3s) of *Haemonchus contortus*, the potency of two active test compounds (MMV1794214 and MMV1794206) on adult females of *H. contortus*, and the potency of one active test compound (MMV1794206) on young adults of *Caenorhabditis elegans*, with reference to monepantel and/or moxidectin (positive controls). Each curve shows (**A**) the inhibition of *H. contortus* larval motility at 90 h, (**B**) the inhibition of *H. contortus* larval development at 7 days, (**C**) the in vitro motility inhibition (%) of *H. contortus* adult females over a period of 24 h (motility scores assessed at 3-, 6-, 12- and 24 h time points; cf. Taki et al. [59]) and (**D**) the reduction of *C. elegans* motility at 40 h. Data points represent either one (**C**) or three (**A**, **B** and **D**) experiments conducted in triplicate; the mean ± standard deviation (SD, **C**) or standard error the mean (SEM, **A**, **B** and **D**). Statistical significance was evaluated with reference to a negative control (**C**); **** denotes *P* ≤ 0.0001

Of the seven compounds that inhibited *H. contortus* xL3 motility by ≥ 70%, three compounds, MMV1577458 (chlorfenapyr), MMV688934 (tolfenpyrad) and MMV0002519 (rotenone), had been previously assessed for motility inhibition on *H. contortus* larvae (cf. [49, 63, 64]). Thus, the remaining four compounds (MMV1794214, MMV1794206, MMV1577463 and MMV027339) were prioritised for further potency assessment on *H. contortus* larval motility inhibition. Following incubation for 90 h (Fig. 2A), MMV1794214 displayed a half-maximal inhibitory concentration (IC_50_) of 4.5 ± 1.1 μM (maximum motility inhibition, MMI: 70%), whereas compound MMV1794206 had an IC_50_ of 18 ± 4 μM (MMI: 98%)—both compounds were less active than the positive-control compound, monepantel (0.33 ± 0.12 μM, MMI: 95%). Of note, compounds MMV1577463 and MMV027339 both displayed motility inhibition (IC_50_) > 40 μM. Subsequently, the potency of MMV1794214 and MMV1794206 to inhibit larval development of *H. contortus* following 168 h of incubation (Fig. 2B) was assessed. MMV1794214 displayed an IC_50_ of > 40 μM, whereas compound MMV1794206 had an IC_50_ of 1.2 ± 0.1 μM, compared to that of monepantel, which displayed an IC_50_ of 0.26 ± 0.03 μM. Both MMV1794214 and MMV1794206 were further evaluated for the inhibition of the motility of adult females of *H. contortus* (at a single concentration of 40 μM; Fig. 2C). While MMV1794214 did not markedly reduce motility (~ 0%, 0%, 23% and 15% at 3 h, 6 h, 12 h and 24 h, respectively), MMV1794206 reduced motility by ~ 33% (3 h), 89% (6 h), 100% (12 h) and 100% (24 h). The monepantel control reduced motility by ~ 0%, 23%, 66% and 100% at 3 h, 6 h, 12 h and 24 h, respectively.

Eight compounds were found to inhibit *C. elegans* young adult motility by ≥ 70%, and one of them (MMV1794206; Fig. 2D) was prioritised for further validation (the remaining seven were already known nematocides). Following incubation for 40 h, MMV1794206 exhibited an IC_50_ of 0.22 ± 0.09 μM (MMI: 100%). In comparison, the monepantel control displayed an IC_50_ of 0.03 ± 0.01 μM (MMI: 95%).

**Fig. 3:**
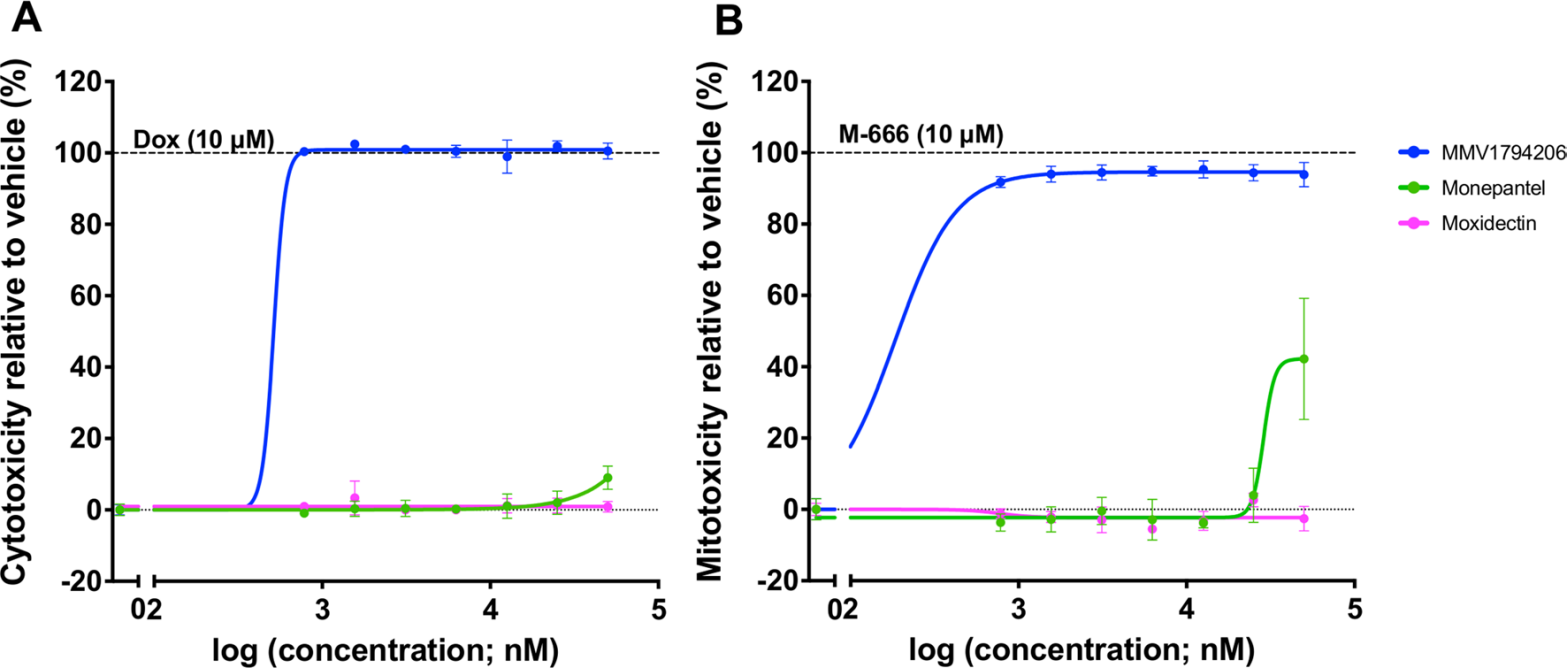
Toxicity assessment of MMV1794206, monepantel and moxidectin on human hepatoma (HepG2) cells, with reference to two positive controls, doxorubicin (Dox; cytotoxic) and M-666 (mitotoxic). For each compound, the (**A**) half-maximal cytotoxic concentration (CC_50_) and (**B**) half-maximal mitotoxic concentration (MC_50_) were established via a cell viability assay after 48 h of incubation. Crystal violet staining was used to measure the absorbance (595 nm) of treated cells, which was negative- (blank) and baseline- (100% cell viability) corrected. Data points represent triplicates and are presented as a mean ± standard deviation (SD)

Given that compound MMV1794206 displayed significant activity on *H. contortus* larvae and adult worms as well as *C. elegans* young adults, its toxicity was assessed by measuring cell death using crystal violet staining. This compound was shown to be both cytotoxic (CC_50_ < 0.7 μM; Fig. 3A) and mitotoxic (MC_50_ < 0.7 μM; Fig. 3B) against HepG2 (human hepatoma) cells.

## Discussion

The screening of the Global Health Priority Box, a collection of 240 molecules with activity against drug-resistant malaria, neglected tropical diseases or vector species, revealed and/or reidentified several compounds with in vitro activity on *H. contortus* and/or *C. elegans*.

Compounds with previously identified activity on *H. contortus*—chlorfenapyr (MMV1577458) [64], tolfenpyrad (MMV688934) [49] and rotenone (MMV0002519) [63]—were all reidentified as hit compounds in this study. Two compounds—MMV1577463 and MMV027339—were initially identified as hits (83 and 72% motility reduction, respectively), but upon further potency evaluation were found to display IC_50_ values (against *H. contortus*) of > 40 μM. Of note, two compounds, MMV1794214 and MMV1794206, were confirmed as inhibitors of *H. contortus* larval motility (IC_50_ values of 4.5 and 18 μM, respectively), with MMV1794206 also showing a significant reduction of larval development and motility of adult females of *H. contortus *in vitro. Although monepantel was more potent at reducing larval motility and development, compound MMV1794206 inhibited the motility of adult females (100% at 12 h) before monepantel did (66% and 100% at 12 and 24 h, respectively).

Of the eight compounds identified as hits based on motility reduction in *C. elegans*, seven—i.e. chlorfenapyr (MMV1577458), moxidectin (MMV1633828), ivermectin (MMV672931), selamectin (MMV002231), milbemectin (a mixture of milbemycin A_3_ and milbemycin A_4_; MMV1578924), abamectin (MMV1577454) and eprinomectin (MMV1633829)—had been investigated previously for antiparasitic activity and/or are commercially available nematocides. Ivermectin, selamectin, abamectin and eprinomectin are all well-known macrocyclic lactones belonging to the chemical family of avermectins [65]; moxidectin and milbemectin are milbemycin macrocyclic lactones [66]. One compound, MMV1794206, was also identified as a hit against *C. elegans*, and validated as a potent motility inhibitor with an IC_50_ of ~ 0.22 μM, compared with 0.03 μM for monepantel. However, MMV1794206 was shown to be toxic to a human cell line (HepG2 cells), suggesting likely adverse effects in mammals administered with this compound.

**Fig. 4:**
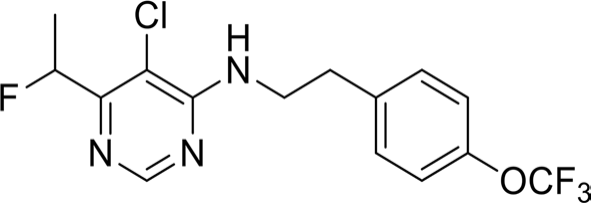
The chemical structure of MMV1794206 (flufenerim)

The potent effect of MMV1794206—herein referred to as ‘flufenerim’—against most of the developmental stages of *H. contortus* (see [67]), and the activity on the related free-living worm *C. elegans*, indicates that this compound is a potential candidate for nematocide development—however, the demonstrated cell toxicity to HepG2 cells presents a barrier for future development. Structurally, flufenerim (Fig. 4)—a 4-aminopyrimidine derivative—is primed for a diverse range of structural alterations via a concise synthetic route [68]. Thus, a structure–activity relationship investigation of flufenerim would be both feasible and cost-effective, but would need to focus on both the optimisation of anthelmintic activity and the elimination of toxicity to mammalian cells, a significant challenge.

Flufenerim has been previously developed as a pesticide against agricultural pests, including aphids (*Aphis medicaginis* and *Myzus persicae*), a fungus (*Pseudoperonospora cubensis*), moths (*Mythimna separata* and *Spodoptera littoralis*) and whiteflies (*Bemisia tabaci*) [68–70]. The mode of action of flufenerim is unclear. Ghanim et al. [69] showed a decrease in acetylcholine esterase (AChE) activity in whiteflies (in vitro and in vivo) following treatment with flufenerim, but it was not confirmed whether this was a result of a direct or indirect effect; Liu et al. [68] reported a similar decrease in AChE activity in aphids. Notably, one drug, naphthalophos (an organophosphate), which inhibits nematode AChE activity was commercially available (in Australia) for use in livestock (as an anthelmintic). Although relatively rare, resistance to naphthalophos in parasitic worms has been reported [71, 72], yet the use of organophosphates is unwarranted due to a relatively narrow safety margin [73]. In contrast, although the Insecticide Resistance Action Committee (IRAC) has not assigned flufenerim’s mode of action, another structurally related acaricide, pyrimidifen, is classified as a mitochondrial complex I electron transport inhibitor (METI) [74]. The conserved 5-chloropyrimidin-4-amine motif does suggest that pyrimidifen and flufenerim share a similar mode of action, namely, as METIs. Several METI insecticides have been previously identified to display anthelmintic activity—notably, the pyrazole compounds tebufenpyrad and tolfenpyrad [75], and the rotenoid compound, rotenone [64]. In addition, a preliminary study [75] suggested that tolfenpyrad may indeed disrupt or interrupt mitochondrial function in *H. contortus*. However, tolfenpyrad was further shown to inhibit respiration in rat hepatoma (FAO cell line) cells, with the level of inhibition of oxygen consumption in FAO cells being positively correlated with compound-induced murine toxicity [76]. Tolfenpyrad had also been previously developed and commercialised as an acaricide ear-tag (for use in cattle; Tolfenpro^®^), but was recalled due to ocular inflammation associated with treatment [77]. Thus, in the case of tolfenpyrad and other METI parasiticides, concerns of mammalian toxicity associated with drug treatment have prevented their development. Indeed, given the high toxicity associated with flufenerim, it will be critical for future compound optimisation and lead development efforts to include rigorous toxicity testing.

If flufenerim were to achieve anthelmintic action via AChE inhibition, mitochondrial interruption and/or another yet unknown pathway, significant laboratory-based target identification and validation experiments would need to be conducted. Importantly, a significant delineation in structure between the nematode and mammalian protein target(s) would be critical in achieving selectivity for the parasite. Anthelmintic target identification and validation (in parasitic species and the free-living *C. elegans*; cf. [45]) has been achieved via competition assays [78–81], electrophysiological studies [82, 83], resistance studies [18, 84–87] and, more recently, RNA interference (RNAi) (e.g., [88]). Recent technological advances have also paved the way for mass spectrometry-based investigations of protein–drug interactions (reviewed by [47, 48]). Methods such as thermal proteome profiling (TPP) have been utilised to identify protein targets for several therapeutics [89–94]—importantly, this method has been used to uncover candidate drug targets of novel nematocides in *H. contortus* (see [95]). Such an approach could also be applied to flufenerim, possibly identifying the drug–protein interactions in this and related nematodes, and providing insight into the anthelmintic mode of action of this candidate.

## Conclusion

To address the global socioeconomic impacts of widespread drug resistance in parasitic nematodes of livestock animals, new anthelmintics with novel modes of action are needed. Using two model nematodes, *H. contortus* and *C. elegans*, we screened a small collection of chemical entities, the Global Health Priority Box, for nematocidal and nematostatic activity. We identified one compound, MMV1794206 (flufenerim), which displayed in vitro anthelmintic activity against key developmental stages of the parasitic worm *H. contortus*, and also inhibited the motility of the related, free-living worm *C. elegans*. However, MMV1794206 was also shown to be toxic to a mammalian cell line (HepG2). Future work should focus on the identification and validation of flufenerim–nematode protein interactions and on undertaking a structure–activity relationship investigation to both optimise anthelmintic activity and eliminate mammalian cell toxicity.

## Data Availability

All data generated and analysed during this study are included in this published article.

## Abbreviations

AChE: Acetylcholine esterase
DMSO: Dimethyl sulfoxide
L3: Third-stage larvae
xL3: Exsheathed third-stage larva
L4: Fourth-stage larva
METI: Mitochondrial complex I electron transport inhibitor
MMI: Maximum motility inhibition
